# Current Perspectives Regarding Stem Cell-Based Therapy for Liver Cirrhosis

**DOI:** 10.1155/2018/4197857

**Published:** 2018-01-29

**Authors:** Kyeong-Ah Kwak, Hyun-Jae Cho, Jin-Young Yang, Young-Seok Park

**Affiliations:** ^1^Department of Oral Anatomy, School of Dentistry, Seoul National University and Dental Research Institute, Seoul, Republic of Korea; ^2^Department of Preventive and Social Dentistry, School of Dentistry, Seoul National University and Dental Research Institute, Seoul, Republic of Korea; ^3^Department of Dental Hygiene, Daejeon Institute of Science and Technology, Daejeon, Republic of Korea

## Abstract

Liver cirrhosis is a major cause of mortality and a common end of various progressive liver diseases. Since the effective treatment is currently limited to liver transplantation, stem cell-based therapy as an alternative has attracted interest due to promising results from preclinical and clinical studies. However, there is still much to be understood regarding the precise mechanisms of action. A number of stem cells from different origins have been employed for hepatic regeneration with different degrees of success. The present review presents a synopsis of stem cell research for the treatment of patients with liver cirrhosis according to the stem cell type. Clinical trials to date are summarized briefly. Finally, issues to be resolved and future perspectives are discussed with regard to clinical applications.

## 1. Introduction

Liver fibrosis results from sustained injury, which can be inflicted by various factors such as viruses, drugs, alcohol, metabolic diseases, and autoimmune attacks [1]. Prolonged exposure to these harmful factors causes hepatocyte apoptosis, inflammatory cell recruitment, endothelial cell impairment, and, lastly, activation of hepatic stellate cells, the major cells involved in liver fibrosis. Liver fibrosis is a kind of scar tissue formation in response to liver damage [2–9]. Histologically, it is caused by an imbalance between extracellular matrix synthesis and degradation [10–12].

Liver cirrhosis is a condition where scar tissue replaces the healthy tissue of the liver and regenerative nodules with surrounding fibrous bands develop as a result of the injury [13]. Cirrhosis is the common end of progressive liver disease of various causes, resulting in chronic liver failure entailing complications such as hepatic encephalopathy, spontaneous bacterial peritonitis, ascites, and esophageal varices [14]. Unfortunately, the majority of cases are usually in an irreversible state when diagnosed. Despite current advancements in its management [15, 16], cirrhosis was the 14th leading cause of death worldwide in 2012 [17]. Orthotopic liver transplantation is known to be the only definite solution to end-stage cirrhosis.

However, several problems preclude the prevalent application of the procedure, including immunological rejection and the scarcity of donor sources [18].

In fact, the liver has an inherent regenerative capacity to a substantial degree [19], and, thus, the cessation of those harmful factors may prevent further progression of fibrosis and reverse the situation in some cases [20]. In cases where hepatocyte proliferation is insufficient for recovery from liver injury, bipotent resident liver progenitor cells (LPC) are activated and participate in liver regeneration by differentiating into hepatocytes and biliary epithelial cells [19, 21–23]. However, fibrosis is inevitable when regeneration is exceeded by destruction. Clinical signs of liver failure usually appear after about 80 to 90% of the parenchyma has been destroyed.

Hepatocyte transplantation has been proposed as an alternative approach to transplantation, since hepatocytes have been proven to be strongly associated with liver repair [24–28]. While hepatocyte transplantation is safe in humans, its applicability remains limited due to organ availability, failure of donor engraftment, weak viability in cell culture, and vulnerability to cryopreservation damage [25, 26, 29–32].

Instead of hepatocytes, the transplantation of stem cells has shown therapeutic potential for liver function improvement according to recent experimental studies and human studies [20, 26, 33–40]. Although they remain unclear, the major potential mechanisms have been proposed as a twofold; one is the improvement of the microenvironments through paracrine effects, and the other is the replacement of functional hepatocytes [20].

To date, several kinds of stem cells have been investigated for their therapeutic feasibility and clinical potential in liver cirrhosis [41–43]. The present article briefly reviews the current literature according to the types of stem cells and discusses the future perspectives of stem cell-based therapy in liver cirrhosis.

## 2. Sources of Stem Cells

Hepatocytes obtained via autopsy of patients who received bone marrow transplantation suggested that they are pluripotent cells in bone marrow [44, 45]. Currently, at least three types of bone marrow-derived cells are known to differentiate into hepatocyte-like cells (HLCs): hematopoietic stem cells (HSCs), mesenchymal stem cells (MSCs), and endothelial progenitor cells (EPCs), though early infusion trials did not discriminate the origins of those cells from bone marrow-derived stromal cells with some improvement [32, 46–52]. A large number of preclinical studies have proven the feasibility of HSCs, MSCs, and EPCs to restore hepatic function in models of liver injury [53–57]. In addition, other stem cells including embryonic stem cells (ESCs) and induced pluripotent stem cells (iPSCs) can also be differentiated into HLCs [58–60]. HLCs can contribute to the remodeling of cirrhotic liver [20, 61–68].

### 2.1. Hematopoietic Stem Cells

HSCs are the predominant population of stem cells within bone marrow and express CD34 as the cell surface marker. They can renew themselves and differentiate into progenitor cells [69, 70]. HSCs can easily be made to leave the bone marrow and circulate into the blood. The mobilization of HSCs resident in bone marrow can be brought about at a low magnitude through tissue injury [71, 72] or in high amounts after artificial priming [73, 74]. Granulocyte-colony stimulating factor is the most widely studied and widely used mobilizing agent [75–80].

HLCs derived from HSCs have been demonstrated to contribute to liver regeneration [65, 81–83]. In general, two mechanisms were proposed with substantial support. One was the de novo generation of hepatocytes through trans-differentiation, and the other was the genetic reprogramming of resident hepatocytes through cell fusion [45, 46, 84]. However, the infused HSCs do not seem to be a primary source of newly generated hepatocytes [85, 86]. Rather, their roles are likely to be associated with macrophages, which produce collagenases, phagocytose dead cells, and facilitate liver regeneration [87–89]. Therefore, the clinical benefit of HSC therapy occurs through paracrine signaling interactions involving various cytokines and growth factors [86, 90, 91]. Furthermore, HSCs likely stimulate neoangiogenesis [92].

### 2.2. Endothelial Progenitor Cells

EPCs are immature endothelial cells that can be found in both peripheral blood vessels and bone marrow. They arise from hemangioblasts and participate in the neovascularization of damaged tissue throughout the whole body [93–99]. Due to their common expression of CD34, EPCs and HSCs are assumed to have a common precursor [94, 100–106]. However, EPCs are likely to be differentiated from various cell lineages, as evidenced by their diverse surface markers [102, 104, 107–113].

The transplantation of EPCs led to the suspension of liver fibrosis by suppressing activating HSCs, according to an animal study [55]. They also promoted hepatocyte proliferation and increased matrix metalloproteinase activity [114]. These effects were associated with increased secretion of growth factors [115–117].

### 2.3. Mesenchymal Stem Cells

MSCs are a rarer population in bone marrow compared to HSCs, which are capable of self-renewal and differentiation into HLCs as well as cell types of mesenchymal origin [68, 118–128]. Traditionally, MSCs have frequently been isolated from bone marrow [129], but, recently, they have been obtained from many other tissues including umbilical cord blood, adipose tissue, and placenta [130–142]. There seems to be source-dependent differences among MSCs [143].

As a therapeutic advantage, MSCs can easily be expanded ex vivo without losing their differentiation potential, and they can also be migrated to the injured areas in response to homing signals [1]. Furthermore, the MSCs have immunomodulatory properties [144–156], through both adaptive and innate immune systems [157, 158], and secrete a variety of trophic factors such as growth factors and cytokines beneficial for liver regeneration [159–164]. Some of these trophic factors are known to revive hepatocytes reaching their replicative senescence [38, 165, 166]. With these advantages as a cell therapy source, the MSCs are the most widely studied stem cells, both experimentally and clinically [59, 167–174].

The precise therapeutic mechanisms of MSCs in liver regeneration have yet to be sufficiently elucidated. Accumulating evidence strongly supports the inference that the effects of MSCs are mediated mostly via paracrine mechanisms rather than transdifferentiation [175–179], although the infused bone marrow-derived MSCs (BM-MSCs) have been shown to engraft into host livers and ameliorate fibrosis in experimental animal models of liver fibrosis [54, 180–183]. MSC transplantation has also demonstrated preclinical efficacy in mitigating liver fibrosis as in other organs [53, 54, 181, 184–187]. Strategies to enhance the effects of MSC in cirrhosis have been investigated, including the facilitation of transdifferentiation into functional hepatocytes [59, 68, 120, 168, 188]. Interestingly, however, the in vivo transdifferentiation of MSCs into hepatocytes has been rarely observed in animal models [54, 189–192]. Rather, the MSCs downregulate proinflammatory and fibrogenic cytokine activity, stimulate hepatocellular proliferation, and promote collagen degradation by matrix metalloproteinase [53, 54, 181, 190–193]. The paracrine effects modulate the functioning of activated hepatic stellated cells [56, 194, 195]. Evidence from treatment with MSC-conditioned medium reconfirmed the paracrine effects of MSCs such as the increased proliferation and reduced apoptosis of hepatocytes subsequent to the upregulation of several anti-inflammatory and antifibrotic cytokines [196, 197]. How each of these signaling molecules individually contributes to hepatic regeneration, however, remains to be further elucidated [46].

### 2.4. Embryogenic Stem Cells

Thomson et al. derived and characterized human ESCs from the inner mass of a blastocyst for the first time in 1998 [198]. ESCs have pluripotency and can differentiate into hepatocyte-like cells, which possess some properties of mature hepatocytes [199–205]. Hepatocytes generated from ESCs in vitro and hepatocytes differentiated from ESCs have been demonstrated to express a number of hepatocyte-related genes and mimic hepatic functions. ESC-derived hepatocytes bear the typical morphology of mature hepatocyte and colonized liver tissue upon transplantation. The cardinal pathways associated with activin A and Wnt3a and FGF signaling are essential for ESC to differentiate into hepatic lineage [200, 206–217]. ESC-derived hepatocyte-like cells promoted the cell recovery of injured liver by cell replacement [201, 210, 218, 219] and paracrine mechanism to stimulate endogenous regeneration. However, it still remains unclear whether ESCs-derived hepatocytes have the origin of definitive endoderm or primitive endoderm. The recent study using ESCs combined with MSCs showed promising results [201, 220]. Human ESCs are likely resistant to cryopreservation, which mature hepatocytes can hardly endure. Studies using ESCs have provided the molecular basis of hepatocyte differentiation. Despite the promising results, the application of human ESCs has always been precluded by practical and ethical barriers.

### 2.5. Induced Pluripotent Stem Cells

The iPSCs were first developed by Dr. Yamanaka from mouse fibroblasts in 2006, which were reprogrammed into a state of pluripotency like that of ESCs [98]. These iPSCs have been reported to be differentiated to neuron cells [221], neurospheres [222], cardiomyocytes [223–225], hematopoietic and endothelial cells [226], and insulin-secreting islet-like clusters [227]. A number of protocols to differentiate iPSCs into HLCs have been described [60, 228–242]. Unfortunately, the iPSC-derived HLCs showed minimal activity, reaching around 0.3 to 10% of the activity of primary hepatocytes [231].

In animal experiments, the iPSC-derived HLC transplantation halted lethal fulminant hepatic failure, promoted regeneration, and improved function [234, 235, 243–245]. Due to immunosuppression and possible unlimited supply, the patient-corrected human iPSCs have great potential to be utilized in personalized cell therapy [230, 246, 247]. However, several issues regarding iPSC usage should be properly addressed prior to clinical application, including teratoma formation and tumorigenicity, controversy about immunogenicity, long-term safety and efficacy, and optimal reprogramming and manufacturing processes [248–250].

### 2.6. Other Cells

Fetal hepatic progenitor cells have been of interest due to their ease of isolation, high proliferation rate, superior repopulation capacity, lower immunogenicity, and resistance to cryopreservation in contrast to adult counterparts [251–256]. Annex stem cells derived from umbilical cord, placenta, and amniotic fluid have easily accessible sources, but they can be categorized as MSCs with respective differences according to their origin [257–261].

## 3. Clinical Trials Using Stem Cell-Based Therapy

Early autologous bone marrow-derived stem cell transplantation resulted in amelioration of liver injury and functional improvements, and they probably included a mixed cell population of HSCs, MSCs, and EPCs [53, 55, 82, 124, 262, 263]. A number of single-arm, phase I clinical studies with small samples have been performed and have shown some promise in patients with liver cirrhosis [31, 48, 49, 123, 264–270]. The infusion of bone marrow-derived stem cells has sometimes been used as a supportive measure for patients with partial hepatectomy [271–273]. Although the precise mechanisms are still unresolved, the findings from those studies with small sample sizes have provided assurance that no critical complications occurred after the procedures. Furthermore, the posttransplantation incidence of hepatocellular carcinoma was not increased despite the enduring concern [68, 274, 275].

A trial using human fetal liver-derived stem cells enrolling 25 patients with cirrhosis demonstrated improved mean model for end-stage liver disease (MELD) scores [276], although long-term outcomes were not properly reported [277]. There have been several clinical studies using HSCs with promising results [278–281] since Pai et al. [37] reported that the autologous infusion of CD34+ cells improved the serum albumin level and the Child-Pugh score. However, most results have shown only temporary effects and there still remains many questions yet to be answered [280].

The most frequently studied stem cells are the MSCs; thus, their mechanisms of actions are also better understood. In particular, BM-MSCs have been prevalently utilized. In two early pilot studies, autologous injections of BM-MSCs in a few patients were reported to result in improvement of liver function [35, 267]. The safety and short-term efficacy of BM-MSCs were evidenced in two groups of 20 patients each, which showed significantly improved Child-Pugh and MELD scores [47]. Subsequent studies continued to confirm the efficacy of BM-MSC transplantation in varying sizes of samples [282–285]. Notably, one randomized controlled trial using autologous MSCs in cirrhotic patients failed to demonstrate beneficial effects, in contrast to the prior reports [286].

The transplanted cells were mostly infused intravenously, except in three studies using the hepatic artery [284, 285] and one featuring direct injection into the spleen [282]. Not a small variation existed in the numbers of infused cells and the administration frequencies. Although the overall study qualities did not surpass the level of moderate or poor, the results seemed promising in terms of MELD scores and liver function improvements [1]. Specifically, most studies did not include histologic evaluations [287].

As other kinds of MSCs, umbilical cord-derived MSCs (UC-MSCs) were evaluated in clinical trials. UC-MSC infusion was well tolerated and resulted in significant functional improvement and increased survival rates [288–290].

To summarize, stem cell trials in patients with liver cirrhosis have demonstrated generalized functional improvements. In addition, improvements were also found in the MELD and Child-Pugh scores. Unfortunately, these beneficial effects were attenuated with time or were not measured. Therefore, it can be temporarily concluded that treatment using stem cells might be slightly superior to current conventional treatment according to two systematic reviews [1, 42] (Table 1).

## 4. Discussion

Liver cirrhosis is a major cause of mortality and incurs great healthcare burdens across the world [291–294]. Liver transplantation is the only effective treatment. The survival rate after liver transplantation has progressively increased and the rate of survival after one year of surgery is currently 83% after one year. However, the shortage of organs is a serious problem contributing to the increasing mortality rate of patients on the waiting list [295, 296]. Allogeneic hepatocyte transplantation [297, 298] also entails limited availability with only modest benefits reported [26, 299, 300].

Efforts have been made to develop antifibrotic therapies. Unfortunately, there are no antifibrotic drugs available in a current clinical setting [301–303] even if several reports have been published from preclinical and clinical studies [304–306]. The targets of the drug are primarily associated with the activities of hepatic stellate cells: the downregulation of cell activation [307–310], neutralization of fibrogenic and proliferative cell responses [311–313], promotion of cell apoptosis [314], and promotion of matrix degradation [315, 316]. Clinical studies have, however, failed to yield meaningful results compared with preclinical studies [287, 317–319].

In this regard, stem cell-based therapy is considered a promising therapeutic alternative based on the discrepancy between the demand and supply of donor livers for transplantation. Stem cell clinical trials have resulted in promising outcomes [20, 209, 230, 246, 277, 320–325]. There are advantages and disadvantages depending on which source of stem cells is used in the cell-based therapies. For example, ethical issues and behavioral uncertainties in vivo are major problems of ESCs or iPSCs to be used clinically although they are the most capability of producing HLCs [20]. Teratoma formation and the use of immunomodulatory drugs are other concerns of stem cell uses. For all kinds of stem cell-based therapies, the progressive liver fibrosis and hepatocellular carcinoma are still the fearful medium- or long-term adverse effects. Prior to clinical use, the in vivo safety should be confirmed including toxicity and tumorigenicity. Regulatory challenges and financial burden cast somewhat different kinds of translational barrier.

Among stem cells of various origins, MSCs have attracted attention due to their advantages and have been extensively investigated in experimental studies and in clinical trials. Nevertheless, there are still a number of issues to be addressed. First, the ideal delivery route of MSCs has not been elucidated, and it is unstandardized in clinical trials to date. MSCs differentiate into myofibroblasts instead of hepatocytes depending on the injection route [326, 327]. The optimal dose and number of injections are another practical issue when comparing the results from clinical trials. In addition, sophisticated methods of tracking engrafted MSCs are still lacking. Therefore, it is impossible to predict the fate of transplanted cells, although the survival duration is important for sustained efficacy [328–330]. Recently, labeling cells with superparamagnetic iron oxide nanoparticles and reporter genes have been suggested with advanced imaging technologies [331–336]. Finally, the quality of the clinical studies reported to date is far from sufficient to reach a definite conclusion. Patient enrollment must differentiate clearly between patients with compensated cirrhosis and patients with impaired function. Only randomized controlled designs can assess the reliable clinical benefit. Long-term follow-up and histologic evidence should be recommended in cases where they are available [42, 250, 337].

With advances in novel biotechnology, strategies have been devised to enhance the effects of stem cell-based therapy. For example, the microencapsulation of MSCs in microspheres was proposed to evade unwanted differentiation into myoblasts [338]. To promote the homing of MSCs, the use of MSCs modified by liver-specific receptors has been suggested [339]. Genome editing using CRISPR/Cas9 is a very promising technology widely used in current functional genomics [18, 340, 341]. The three-dimensional culture technique is another example for providing an expansion and differentiation platform for hepatocytes [342, 343].

Rapidly developing iPSC technologies provide an unprecedented opportunity for researchers and clinicians [344]. Recent studies have shown that iPSC-derived hepatocytes can be used for the investigation of the genetic and molecular mechanisms of liver disorders [240, 242, 244, 345–356]. They can be utilized for multiple applications, including drug safety screening of new drugs [214, 357–359] and disease modeling [240, 360]. Disease-specific iPSCs could provide invaluable opportunities to elucidate the pathologic mechanism of disease and develop curative treatment options.

## 5. Conclusion

Liver fibrosis progresses to cirrhosis, which is the result of the extracellular matrix deposition in the parenchyma. Curative treatment for cirrhosis is currently limited to orthotopic liver transplantation, and a worldwide shortage of donor organs results in the deaths of patients waiting for organs. Stem cell-based therapy has emerged as a promising alternative with accumulating evidence from experimental and clinical studies. Varieties of stem cells including MSCs, HSCs, EPCs, ESCs, and iPSCs have been investigated for their feasibility and/or clinical potentials. Among them, MSCs have been most studied and are relatively well understood. A primary mechanism of action has been proposed as paracrine effects rather than transdifferentiation. The results from clinical trials seem very promising from the perspectives of functional improvement and clinical parameters. However, long- term efficacy has not yet been proven, and standardized trial protocols are needed. Novel technologies are expected to overcome the current hurdles related to clinical application of stem cell-based therapy.

## Figures and Tables

**Table 1:** Main clinical trials of stem cell therapy for liver cirrhosis.

Trial number	Study phase (type)	Cell source	#	Eligibility criteria	Primary outcome measure	Secondary outcome measure	Time frame	Start date	End date	Location
NCT01875081	Phase II (randomized open)	BM-MSC	72	Histologically or clinically diagnosed as alcoholic liver cirrhosis Classified as Child-Pugh grade B or C	Histopathological evaluation	Histopathological evaluation score, MELD score, Child-Pugh grade, and so on	6 months	2012.11	2016.03 (completed)	Korea

NCT02943889	Phase I/II (nonrandomized open)	BM-MSC	40	Decompensated liver cirrhosis Child class b or c	Improvement of liver function in form of improvement in Child score	Postpone or overcome liver transplantation complications	6, 24 months	2016.10	2017.08 (not yet recruiting)	None

NCT02786017	Phase I/II (randomized double-blinded controlled)	UC-MSC	40	Subjects who are decompensated cirrhosis of any cause Child-pugh score ≥7	Change in the model for end-stage liver disease (MELD) score	Change in Child-Pugh score, clinical laboratory parameters of liver function	1 and 3 days 1 and 2 weeks 1, 3, 6, 12, and 24 months	2016.05	2018.12 (recruiting)	China

NCT01591200	Phase II (randomized open)	AlloMSC	40	Child class B or C, Child-Pugh scores of ≥7 and <14 MELD scores of at least 10	Safety	Liver function improvement, Child-Pugh score, MELD score, SF36-QOL, and so on	24 months	2012.06	2016.04 (completed)	India

NCT01120925	Phase I/II (randomized quadruple blind controlled)	BM-MSC	30	MELD score of 12 or Child score B or C Serum ALT 1/5 times more than normal	Liver function test	Cirrhosis mortality	6 months	2010.05	2013.07 (completed)	Iran

NCT00420134	Phase I/II (randomized single- blinded)	MSC	30	MELD score of at least 10 Patent portal vein on color Doppler examination of the live Normal alpha-feto protein serum levels	Liver function test MELD score	Cirrhosis mortality	6 months	2006.02	2009.06 (completed)	Iran

NCT01013194	Phase I/II (nonrandomized open)	FLC	25	A score ≥ B8 based on the Child-Pugh-Turcotte classification and/or MELD score ≥ 14	Survival	Analysis of Child-Pugh score, meld score from baseline to 1-year follow-up	6 and 12 months	2007.02	2011.07 (completed)	Italy

NCT01342250	Phase I/II (randomized open)	UC-MSC	20	Decompensated liver cirrhosis, Child-Pugh B/C (7–12 points) or Meld score ≦ 21.	Survival	Liver function improvement, Child-Pugh score, MELD score, SF36-QOL, and so on	24 months	2010.10	2011.10 (completed)	China

NCT02652351	Phase I (open)	UC-MSC	20	Clinical, radiological, or biochemical evidence of liver cirrhosis	Severity of adverse events	Hepatic function, liver fibrosis index	1, 3, 6, and 12 months	2016.03	2016.10 (recruiting)	China

NCT01147380	Phase I (nonrandomized open)	NK	18	Subjects who need to meet the liver transplant eligibility criteria Cardiac and pulmonary function	Side effect of cadaveric donor liver NK cell infusion	NK cell infusion-related toxicity, anti-HCC, HCV effect	12 and 24 months	2010.06	2014.12 (completed)	USA

NCT03254758	Phase I/II (open)	AD-MSC	15	Chronic hepatitis C or nonalcoholic steatohepatitis (NASH) Child-Pugh grade B liver cirrhosis	Child-Pugh score, safety profile	Child-Pugh score, safety profile	6 months	2017.07	2018.12 (recruiting)	Japan

NCT01333228	Phase I/II (open)	BM-EPC	14	Liver cirrhosis (Child-Pugh 8 or above)	Safety and tolerability	Effect on liver function, portal hypertension, complications of liver cirrhosis	12 months	2012.06	2015.03 (completed)	Spain

NCT01503749	Phase I (randomized open)	PB-MNC (G-CSF)	9	Advanced liver cirrhosis with Child-Pugh score 8 or 9	Severe adverse events	Change in Child-Pugh score and MELD score	1–4 weeks 2–6 months	2012.01	2014.08 (completed)	-

NCT00713934	Phase I/II (randomized single- blinded)	BMMNC BMHSC	7	Liver biopsy showing histological cirrhosis, grade B or C (Child-Pugh score) liver cirrhosis in sonography study Liver crrhosis in sonography study	Liver function test MELD score	Cirrhosis mortality	6 months	2008.01	2009.02 (completed)	Iran

NCT02297867	Phase I (open)	ADSC	6	Investigators without HBV, HCV, HIV, syphilis, and so on	MELD	None	1–6 months	2015.07	2018.01 (active, not recruiting)	Taiwan

NCT02705742	Phase I/II (open)	AD-MSC	5	Clinical, radiologic, and pathologically proven liver cirrhosis due to HCV hepatitis	All cause mortality	-	12 months	2016.01	2017.12 (recruiting)	Turkey

NCT01454336	Phase I (open)	BM-MSC	3	Approved cirrhosis by elastography, biopsy, sonography	ALT, AST, serum albumin, liver fibrosis	Progression of fibrosis	12 months	2010.06	2013.07 (completed)	Iran

^#^Number of enrollments; MELD, model for end-stage liver disease; UC-MSC, umbilical cord mesenchymal stem cell; AlloMSC, allogeneic MSC; FLC, fetal liver cell; BM-EPC, bone marrow-derived endothelial progenitor cells; PB-MNC, peripheral blood mononucleated cells; BMHSC, bone marrow CD133+ hematopoietic stem cell.
